# Chemical Composition and Antioxidant, Antimicrobial, and Antiproliferative Activities of *Cinnamomum zeylanicum* Bark Essential Oil

**DOI:** 10.1155/2020/5190603

**Published:** 2020-04-29

**Authors:** Behrooz Alizadeh Behbahani, Fereshteh Falah, Fahimeh Lavi Arab, Moones Vasiee, Farideh Tabatabaee Yazdi

**Affiliations:** ^1^Department of Food Science and Technology, Faculty of Animal Science and Food Technology, Agricultural Sciences and Natural Resources University of Khuzestan, Mollasani, Iran; ^2^Department of Food Science and Technology, Faculty of Agriculture, Ferdowsi University of Mashhad, Mashhad, Iran; ^3^Immunology Research Cente, BuAli Research Institute, Faculty of Medicine, Mashhad University of Medical Sciences, Mashhad, Iran; ^4^Department of Anesthesiology, School of Medicine, Yazd Branch, Islamic Azad University, Yazd, Iran

## Abstract

This study examines the chemical constituents, antioxidant potential, antibacterial mechanism, and antiproliferative activity of *Cinnamomum zeylanicum* bark essential oil. The compositions of the oil were analyzed by GC-MS, and the major constituents were found to be (*E*)-cinnamaldehyde (71.50%), linalool (7.00%), *β*-caryophyllene (6.40%), eucalyptol (5.40%), and eugenol (4.60%). *C. zeylanicum* essential oil contained remarkable levels of phenolic and bioactive compounds with outstanding ability to scavenge free radicals and inhibit *β*-carotene oxidation. The growth of pathogenic and spoilage bacteria, especially Gram-positive ones (i.e. *Listeria innocua*, *Staphylococcus aureus*, and *Bacillus cereus*), was highly inhibited by the oil, compared to the Gram-negative pairs (i.e. *Escherichia coli*, *Pseudomonas aeruginosa*, and *Salmonella typhi*). The cells of *L. innocua* and *E. coli* (as the most sensitive and resistant strains to the oil, respectively) treated with *C. zeylanicum* essential oil were observed by scanning electron microscopy to unravel structural changes. It was observed that the essential oil quickly exerted its antibacterial activity through disrupting cell envelope and facilitating the leakage of intracellular compounds. The essential oil had also a dose-dependent antiproliferative effect on adipose-derived mesenchymal stem cells (AT-MSCs), and the cell proliferation could be induced by low concentrations of the oil. The present study indicated that *C. zeylanicum* essential oil with remarkable antioxidant and antimicrobial properties could be applied to develop novel natural preservatives for food and medicinal purposes.

## 1. Introduction

Bacteria resistant to antibiotics-derived infections are nowadays considered as one of the largest problems faced by medicine and food industry, due to the requirement for more difficult, cumbersome, and costly processes to diagnose and treat the related severe infections [1].

Currently, many studies have focused on the evaluation of natural products as the source of novel biologically active compounds, owing to the deficiency of common antimicrobial agents to treat infectious diseases [2, 3]. In the last two decades, there has been an increasing attention in the use of medicinal herbs to develop new herbal medicines or nutraceuticals. The herbal medicines are rich in bioactive molecules (e.g., polyphenols, carotenoids, and flavonoids) with therapeutic effects, such as delaying the onset of some diseases like cardiovascular disorders, diabetes, and cancer [4].

Essential oils derived from medicinal herbs have versatile applications in ethnomedicine, cosmetics, food, beverages, preservation, fragrance, and pharmaceutical industries. These bioactive compounds indicate several positive biological properties, such as antioxidant, antiviral, antibacterial, antifungal, insecticidal, and anticancer activities [5].

*Cinnamomum zeylanicum* belongs to the *Lauraceae* family that grows wild in India, Sri Lanka, Indochina, and Madagascar. Its inner bark has been used as a potent therapeutic agent in ethnomedicine and as a flavoring ingredient in foods [6]. The antioxidant, antimutagenic, and antimicrobial activities of *C. zeylanicum* extracts have been previously evaluated [7, 8]. However, to our knowledge and as seen in the literature, there are little published reports unraveling the mechanism behind the antibacterial activity of *C. zeylanicum* bark essential oil against the most resistant and sensitive food-borne pathogenic and spoilage bacteria to the oil. Therefore, the present study aimed to assess the antibacterial activity of *C. zeylanicum* essential oil through a mechanistic approach to provide more information about its antibacterial mechanism. The *in vitro* antiproliferative effect of the essential oil on adipose-derived mesenchymal stem cells (AT-MSCs) was also investigated.

## 2. Materials and Methods

### 2.1. Materials

Six-well flat-bottom plates were supplied from Iwaki SciTech Co. (Japan). Folin–Ciocalteu reagent, *β*-carotene, linoleic acid, Tween 40, gallic acid, and DPPH (2,2-diphenyl-1-picrylhydrazyl) were purchased from Sigma-Aldrich (St Louis, MO, USA). Microbial media Mueller Hinton Broth (MHB) and Mueller Hinton Agar (MHA) were procured from Merck Co. (Darmstadt, Germany).

### 2.2. Essential Oil Isolation

*C. zeylanicum* bark (Mashhad, Iran) was subjected to hydrodistillation extraction technique to isolate its essential oil. Dried bark (20 g) was transferred to a Clevenger-type apparatus, and the extraction process was run for 3 h. The resultant essential oil was collected in clean glass vials, dried by anhydrous sodium sulfate, and stored at 4°C until analyses [6].

### 2.3. Essential Oil Characterization

#### 2.3.1. Gas Chromatography-Mass Spectrometry (GC-MS)

The chemical compounds of *C. zeylanicum* essential oil were identified by a GC coupled with a mass spectrometer (TRACE MS, Thermo Finnigan) with a DB-5 capillary column (30 m × 0.25 mm, 0.25 *μ*m stationary phase thickness). The column temperature was increased from 40°C to 250°C with the heating rate of 2.5°C·min^−1^. Helium was applied as the carrier gas, and the ionization energy was 70 eV. The retention indices and mass spectra of the constituents of the essential oil were compared with the published data in the literature, followed by calculation of the retention indices using a homologous series of n-alkane (C_8_-C_20_) indices [9, 10].

#### 2.3.2. Fourier Transform Infrared Spectroscopy (FTIR) Analysis

The functional groups of the essential oil constituents were analyzed by a FTIR spectrometer. The essential oil was mixed with potassium bromide, and the obtained mixture was compressed to form an appropriate pellet. The FTIR spectrum of the oil was obtained at 500–4000 cm^−1^ with 4 cm^−1^ scanning resolution.

#### 2.3.3. Total Phenolic Contents (TPCs)

The TPC of *C. zeylanicum* essential oil was determined by means of Folin–Ciocalteu reagent based on a method described by Abeysekera et al. [11]. 20 *μ*L of the essential oil was added to 110 *μ*L of freshly prepared Folin–Ciocalteu reagent (10-time diluted) which was followed by adding sodium carbonate solution (70 *μ*L), incubating at ambient temperature for 30 min, and recording its absorbance at 765 nm. Gallic acid (0.06–1 mg·mL^−1^) was used to formulate the standard curve, and the TPC of the essential oil was then expressed as mg gallic acid equivalent (GAE)/g dry weight *C. zeylanicum* bark.

### 2.4. Antioxidant Activity Assays

#### 2.4.1. DPPH Radical Scavenging Activity

An aliquot of 37.5 *μ*L of the essential oil or methanol (control sample) was charged and mixed with 2 mL of the DPPH methanolic solution (10^−4^ M). The samples were incubated in the dark for 20 min followed by measuring their absorbance at 517 nm against methanol. The following equation was used to calculate the DPPH radical scavenging activity of the essential oil [12]:

(1) $$\begin{array}{c} \text{DPPH radical scavenging activity }\left(\%\right)\;=\left(1-\frac{\text{sample absorbance }}{\text{control absorbance}}\right)\times 100. \end{array}$$

#### 2.4.2. *β*-Carotene Bleaching Assay

The procedure of Ribeiro-Santos et al. [10] was used to perform this assay. 1 mL of *β*-carotene solution (2 mg *β*-carotene in chloroform) was mixed with Tween 40 (200 mg) and linoleic acid (20 mg). The chloroform was evaporated by vacuum drying of the mixture at 40°C and then oxygenated ultrapure water (50 mL) was added. Afterwards, the solution was vigorously shaken to form a linoleic acid/*β*-carotene emulsion, which (5 mL) was subsequently incorporated with the essential oil (200 *μ*L). The resultant mixture was heated in water bath for 120 min, and its absorbance was then recorded at 470 nm (A_S120_). The control sample was prepared according to the same procedure, but methanol was used instead of the essential oil. The absorbance of the control sample was also recorded at 470 nm before (A_C0_) and after 120 min (A_C120_) incubation time. The antioxidant activity of the oil was then calculated using the below equation:

(2) $$\begin{array}{c} \text{antioxidant activity }\left(\%\right)=\left(\frac{{\text{AS}}_{120}-{\text{AC}}_{120}}{{\text{AC}}_{0}-{\text{AC}}_{120}}\right)\times 100. \end{array}$$

### 2.5. Antimicrobial Activity

#### 2.5.1. Microorganisms

All lyophilized bacterial strain cultures used in this study were *Staphylococcus aureus* ATCC 25923, *Listeria innocua* ATCC 33090, *Bacillus cereus* ATTC 14579, *Pseudomonas aeruginosa* ATCC 27853, *Escherichia coli* ATCC 25922, and *Salmonella typhi* ATCC 6539. They were subcultured in MHB for 24 h at 37°C under sterile conditions. The obtained stock culture was then subcultured in slant nutrient agar and washed several times with sterile ringer solution to prepare fresh microbial suspension. The optical density (at 630 nm) of the suspension was adjusted to provide a microbial suspension with 0.5 McFarland standard or 1.5 × 10^8^ CFU/mL.

#### 2.5.2. Disc Diffusion Agar Method

The microbial suspension was streaked onto the Petri dishes containing MHA. The essential oil (20 *μ*L) was added to soak the filter paper discs with 6 mm in diameter, and the discs were then placed on the inoculated plates, followed by incubation at 37°C for 24 h. The inhibition zone diameter around filter paper discs was measured in millimeters [6].

#### 2.5.3. Well Diffusion Agar Method

In this assay, the microbial suspension was spread onto MHA medium in Petri dishes using a L-shaped spreader. Afterwards, several wells (6 mm in diameter) were created on the medium surface and loaded with the essential oil (20 *μ*L). The Petri dishes were kept at a constant temperature of 37°C for 24 h, and the diameter of the inhibition zones around the wells was measured and expressed in millimeters [13].

#### 2.5.4. Minimum Inhibitory Concentration (MIC) and Minimum Bactericidal Concentration (MBC)

MIC is defined as the lowest essential oil concentration that results in no visible (no turbidity) bacterial growth, and MBC is defined as the lowest essential oil concentration with initial inoculum bacteria killed (no colony formation) [14]. The method of Behbahani et al. [15] with minor changes was applied to measure the MIC and MBC of *C. zeylanicum* essential oil.

Firstly, the sequential concentrations of the essential oil (100, 50, 25, 12.5, 6.25, 3.125, 1.562, 0.78, and 0.39 mg·mL^−1^) were prepared in MHB medium and sterilized using 0.45 *μ*m syringe filters. Each concentration (200 *μ*L) of the oil was added to the wells (in 96-well plates) which were previously filled with 20 *μ*L of microbial suspensions. After incubation of the plate at 37°C for 24 h, triphenyltetrazolium chloride solution (5%, 20 *μ*L) was added and the plate was reincubated. The lowest concentration of the oil with microbial growth-suppression effect, indicated by the lack of dark-red color in the wells, was considered as the MIC.

One hundred microlitre of the solution in the wells with no microbial growth was then cultured onto the plates containing MHA medium. The plates were subsequently stored at 37°C for 24 h, and the lowest concentration of the oil that killed the bacterial strains, confirmed by the lack of visible colonies on the medium surface, was regarded as the MBC.

### 2.6. Antimicrobial Mechanism of Action

According to the MIC results, *L. innocua* and *E. coli* were selected as the most sensitive and resistant bacteria to the essential oil, respectively, in order to investigate the antimicrobial mechanism action of the oil on their cell membranes. The bacterial strains were cultured in a broth medium containing MIC of the oil for each bacteria, while the medium being shaken at 37°C. The microbial suspension was centrifuged (5000 ×g for 5 min) to separate the bacterial strains, followed by washing the strains twice by 0.1 M sodium phosphate buffer (pH 7) and filtration using polycarbonate filters. Glutaraldehyde solution (2.5% v/v) was then used to fix the filtrate, and the solution was incubated at 4°C for 120 min. Double distilled water was applied to wash the sample, and ethanol (at increasing concentrations of 30%, 50%, 70%, 80%, 90%, and 100%) was successively used to dehydrate the sample for 10 min. Afterwards, the sample was vacuum dried to evaporate ethanol, coated with gold, and analyzed by means of a scanning electron microscopy apparatus (LEO 1450 VP model, Germany) to check the morphology of the bacterial strains upon treatment with the essential oil [16].

### 2.7. Cell Viability Assay

Antiproliferative effect of the essential oil against AT-MSCs was analyzed in 96-well flat-bottom plates using MTT [3-(4,5-dimethylthiazolyl)-2,5-diphenyl-tetrazolium bromide] assay, utilizing the method of Yousefi et al. [17] with some modification. AT-MSCs from healthy donors were obtained and (1 × 10^5^ per well) were seeded in the plates until 50–60% confluence was obtained, followed by replacing the medium with 2 mL of complete culture medium (Dulbecco's Modified Eagle's Medium + fetal bovine serum) and adding different concentrations of the oil (1, 3.125, 6.25, 12.5, 25, 50, 100, and 200 mg·mL^−1^) into each well. The plates were incubated for 24 h, and cell proliferation was then quantified by MTT assay. Each well was loaded with 30 *μ*L of 5 mg·mL^−1^ MTT solution, and the plates were incubated in a CO_2_ incubator for 3 h. The medium was discarded gently, and the wells were charged with 200 *μ*L of dimethyl sulfoxide. The absorbance of the mixture was finally read at 570 nm using an ELISA reader (Convergent Technologies, Marburg, Germany).

### 2.8. Statistical Analysis

All data were processed by one-way analysis of variance (ANOVA) in SPSS software. The results were reported as mean ± standard deviation, and the experiments were replicated three times.

## 3. Results and Discussion

### 3.1. Chemical Compositions of the Essential Oil

Essential oils and their biologically active constituents haveoutstanding antioxidant and antimicrobial properties. Thus, they have been vastly applied in food industry, such as in active packaging and surface sanitation of meat and fresh produce [18]. The present study has therefore evaluated the chemical constituents, antioxidant effect, and antibacterial activity of *C. zeylanicum* essential oil. GC-MS analysis resulted in the identification of 17 chemical compounds for *C. zeylanicum* essential oil, as indicated in Table 1. (*E*)-cinnamaldehyde (71.50%), linalool (7.00%), *β*-caryophyllene (6.40%), eucalyptol (5.40%), and eugenol (4.60%) were the main components of the essential oil. The other important constituents were *p*-cymene (1.90%), *α*-humulene (1.70%), *δ*-cadinene (1.40%), *α*-pinene (1.30%), and limonene (1.20%). In accordance with our results, several studies have reported that cinnamaldehyde is the major chemical compound of *C. zeylanicum* bark essential oil [6, 19].

### 3.2. FTIR Analysis

**Figure 1:**
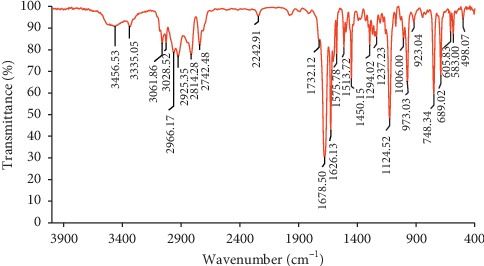
FTIR spectrum of *Cinnamomum zeylanicum* bark essential oil.

Essential oils are considered as complex mixture systems. FTIR spectroscopy was used to identify the functional groups in the essential oil of *C. zeylanicum* (Figure 1). The peaks located at around 1678 cm^−1^ and 1626 cm^−1^ are attributed to the vibration stretching of aldehyde carbonyl (C=O) groups, representing a high concentration of cinnamaldehyde and aldehydes in *C. zeylanicum* essential oil [20]. Other significant peaks were observed at 689 cm^−1^ (vibration absorption of alkanes), 748 cm^−1^ (benzene rings = CH), 973 cm^−1^ (C-H bond), 1124 cm^−1^ (C-O and C-OH bonds), 1237 cm^−1^ (C-O-C bond of aromatic acid ester and C-OH groups of phenolic compounds), 1294 cm^−1^ (alkanes CH2), 1450 cm^−1^ (alcohol C-OH bond), 1450–1626 cm^−1^ (C=C bond), 1575 cm^−1^ (aromatic C=C bond) 1626–1732 cm^−1^ (C=O bond of carbonyl groups), 2814 cm^−1^ (C-H bond of carbonyl groups), 2925 cm^−1^ (=C-H bond), and 3026 cm^−1^ (aromatic C-H bond) [21, 22]. All these characteristic peaks confirm that the essential oil is rich in phenolic and aromatic compounds, especially cinnamaldehyde.

### 3.3. Total Phenolic Content

Phenolic compounds, as a major group of phytochemicals, have great importance owing to their antioxidant activity. The essential oil *C. zeylanicum* had a TPC of 106.19 ± 0.63 mg GAE/g dried essential oil. A TPC of 0.91 [23], 48.90 [24], 53.74 [25], and 912 mg GAE/g [26] was reported for *C. zeylanicum* essential oil. These controversial results suggest that the composition and quality of essential oils from plant sources are strongly influenced by the age and variety of the plant, geographical conditions, drying methods, and the extraction procedures used to isolate the essential oils [27].

### 3.4. Antioxidant Activity

The antioxidant activity of essential oils is most likely attributed to a synergy among their constituents, and the major constituents are primarily responsible for this positive biological effect of essential oils [28]. In this work, DPPH-RS activity and *β*-carotene bleaching assays were used to determine the antioxidant activity of *C. zeylanicum* essential oil.

The DPPH-RS activity assay is based on the reaction of potent antioxidants with DPPH stable free radicals with intense violet color and subsequently converts them to a colorless compound; thus, the discoloration degree indicates the free radical scavenging activity of the antioxidant agent [29]. The DPPH-RS activity of the essential oil of *C. zeylanicum* was found to be 71.12 ± 0.77%, indicating the strong ability of the oil to neutralize DPPH free radicals *via* either hydrogen atom or electron donation mechanisms [30].

The *β*-carotene bleaching assay was employed to evaluate the potential of *C. zeylanicum* essential oil to prevent lipid peroxidation. It is a useful and practical assay due to the fact that it is performed in an emulsion and many food products tend to be emulsified during their productions [31]. The *C. zeylanicum* essential oil had a high inhibitory effect (63.08 ± 0.81%) against *β*-carotene discoloration. This means that the oil is able to scavenge and neutralize linoleate free radicals that are responsible for *β*-carotene discoloration [32]. The present results are in line with the findings of other studies [10, 11], and the antioxidant activity of the essential oil is mainly ascribed to its phenolic and other bioactive compounds. For example, the antioxidant activity of (*E*)-cinnamaldehyde [33], *α*-pinene [34], eugenol [35, 36], *β*-caryophyllene [37], and eucalyptol [38] has been reported in the literature. It is therefore possible to deduce that the *C. zeylanicum* essential oil had strong radical scavenging characteristics along with the potential to suppress lipid oxidation reaction. The oil could be used as a natural alternative to the synthetic antioxidants in the food preservation technologies in order to improve the oxidative stability of many food products.

### 3.5. Antibacterial Activity

The antibacterial activity of *C. zeylanicum* essential oil was investigated towards some pathogenic Gram-positive (i.e. *L. innocua*, *S. aureus*, and *B. cereus*) and Gram-negative (i.e. *S. typhi*, *P. aeruginosa*, and *E. coli*) bacterial species, using disc diffusion agar, well diffusion agar, MIC, and MBC methods. The oil indicated a strong antibacterial effect towards all the microorganisms tested, as indicated in Table 2. It had a bacterial type-dependent antimicrobial activity; the highest and lowest inhibition zones were observed for *L. innocua* and *E. coli*, respectively, according to the results of disc/well diffusion agar tests. Moreover, a greater antibacterial activity or inhibition zone was found in the well diffusion agar compared to the disc diffusion agar assay. Indeed, the bacterial species in the well diffusion agar method are in direct contact to the essential oil, but the diffusion rate of the antimicrobial agent from the disc surfaces to the medium determines its inhibitory effect in the disc diffusion agar test [9, 13].

The MIC and MBC values of *C. zeylanicum* essential oil against the aforementioned bacteria are also reported in Table 2. As can be clearly observed, the Gram-positive bacteria were growth-suppressed or killed in the presence of lower concentrations of the essential oil compared to the Gram-negative ones, mainly due to the presence of a single mucopeptide layer in their cell membranes that makes them to be more sensitive to the antimicrobial agents. In contrast, the cell membranes of Gram-negative bacteria contained a more complexed lipopolysaccharide and phospholipid layer with remarkably lower diffusion rate to lipophilic-based antimicrobial compounds of the essential oils [1, 4].

Similar observations have been previously reported by researchers [6, 39]. The compound cinnamaldehyde, as the main chemical constituent of *C. zeylanicum* essential oil, has been implicated in the antimicrobial effect towards Gram-negative/positive bacteria and fungi species, through inhibiting the formation of essential bacterial enzymes and/or causing intense damage to the bacterial cell walls [40, 41]. Hence, the high antibacterial potential of *C. zeylanicum* essential oil could be attributed to its high cinnamaldehyde concentration.

### 3.6. Antibacterial Mechanism of Action of the Essential Oil

**Figure 2:**
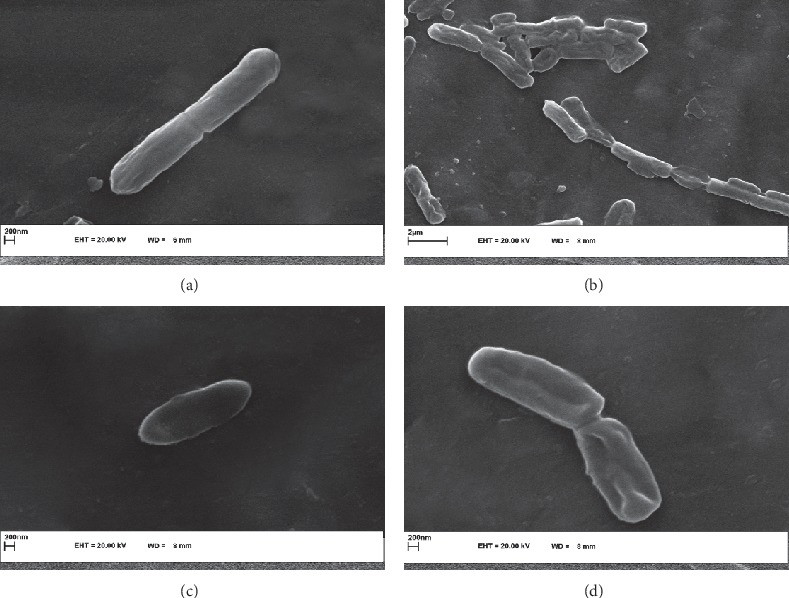
Scanning electron microscopy images of untreated *E. coli* cells (a), treated *E. coli* cells (b), untreated *L. innocua* cells (c), and treated *L. innocua* cells (d) with *Cinnamomum zeylanicum* bark essential oil.

Figure 2shows the microbial cells before and after treatment with *C. zeylanicum* essential oil. The cell appearances were changed upon treatment, likely through altering outer cell envelopes. The untreated *E. coli* cells have their typical striated wall structures (Figure 2(a)), and the differences between the control cells and the treated ones can be easily observed in the rod morphology (Figure 2(b)). It seems that the essential oil of *C. zeylanicum* has the ability to disrupt the membrane of *E. coli* cells and facilitate intracellular compounds leakage, as indicated by the presence of malformed and sunken cell shapes. In fact, the essential oil resulted in a high cell disruption and subsequently greater cell lysis degrees in *E. coli*. The strained wall structures of *L. innocua* cells were changed after treatment with the essential oil, as well (Figures 2(c) and 2(d)). The oil caused deformity in the cell surfaces, and the treated *L. innocua* cells had wrinkled and irregular cellular shapes.

The present results have confirmed the detrimental effect of *C. zeylanicum*-originated essential oils against the cell wall of *E. coli* and *L. innocua*. The corresponding changes in cell walls of bacterial species could be due to the membrane lysis and transformations induced by the damage on the membrane integrity and permeability from the essential oil [14]. It seems that *C. zeylanicum* essential oil could act on the membrane and lead to a marked change in its lipid profile and increase in its surface areas, thereby altering its structure. It also has the potential to penetrate the deeper part of the cells and facilitate their death rate [40].

### 3.7. Antiproliferative Effect

The antiproliferative activity of *C. zeylanicum* essential oil was investigated against AT-MSCs using MTT assay. MSCs, as multipotent self-renewing cells, have the potential to differentiate towards various cell/tissue lineages and produce growth-improving secretomes with antioxidant and anti-inflammatory agents, thereby making them to be used as an appropriate candidate in regenerative medicine [17].

**Figure 3:**
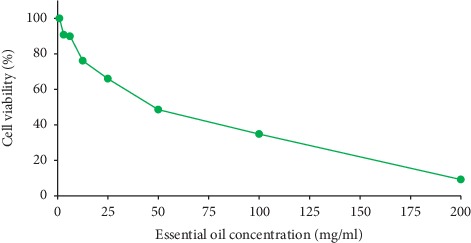
Cytotoxic activity of *Cinnamomum zeylanicum* bark essential oil against adipose-derived mesenchymal stem cells (AT-MSCs) by MTT assay.

The cells were exposed to increasing doses of the essential oil ranging from 1 to 200 mg·mL^−1^ for 24 h. The essential oil had a concentration-dependent growth-inhibition effect on AT-MSCs, and the antiproliferative activity of the oil was increased markedly as its concentration increased up to 200 mg·mL^−1^ (Figure 3). The IC_50_ value for the antiproliferative effect of the oil on AT-MSCs was calculated to be 83.51 mg·mL^−1^. It seems that low concentrations of *C. zeylanicum* essential oil are able to induce cell proliferation and are beneficial for AT-MSC growth, besides having an outstanding antioxidant activity and strong bactericidal effect (at MBC < 50 mg·mL^−1^) against all pathogenic and spoilage bacteria tested in this study.

Essential oils could exert their antiproliferation through several mechanisms including (i) disrupting cell membrane integrity *via* depolarization, permeability increment, or reducing membrane-bound enzymes activity, (ii) altering mevalonate metabolism pathway, or (iii) inducing apoptosis [5]. The antiproliferative effect of *C. zeylanicum* essential oil has been previously shown against different cell lines, such as F2408 (normal rat fibroblasts) and 5RP7 (H-*ras* active-rat fibroblasts) which may be related to synergic effect of some volatile compounds with antioxidant characteristics present in the extract [6].

## 4. Conclusion

The results of the present research showed that the essential oil obtained from *C. zeylanicum* is rich in bioactive compounds, and its main chemical constituent is (*E*)-cinnamaldehyde, comprising 71.5% of the total oil composition. The essential oil conferred superb antioxidant activity and antibacterial effect, especially towards Gram-positive bacteria. A dose-dependent antiproliferative activity was also observed when AT-MSCs were exposed to the essential oil at dosages from 1 to 200 mg·mL^−1^ for 24 h. Results have shown to be very positive and indicated good potential of the oil for use in food products as a natural bioactive ingredient. However, further studies are required to unravel the mechanisms underlying the antiproliferative activity of the essential oil of *C. zeylanicum* and to scale up its application in many food products.

## Figures and Tables

**Table 1:** Chemical constituents of *Cinnamomum zeylanicum* bark essential oil.

Compounds	Retention time (min)	KI	%
*α*-Pinene	5.66	899	1.3
Benzaldehyde	6.40	963	0.3
*p*-Cymene	7.82	1025	1.9
Limonene	7.93	1075	1.2
Eucalyptol	8.08	1084	5.4
*γ*-Terpinene	8.66	1121	0.4
Linalool	9.86	1188	7
Isoborneol	11.64	1275	0.8

*(E)-cinnamaldehyde*	15.22	1414	**71.5**
Eugenol	16.90	1469	4.6
*β*-Caryophyllene	18.58	1518	6.4
Acetic acid, cinnamyl ester	19.23	1536	0.5
*α*-Humulene	19.47	1543	1.7
*δ*-Cadinene	20.97	1581	1.4
*trans*-Calamenene	21.10	1585	0.7
Caryophyllene oxide	22.61	1621	0.5
Benzyl benzoate	26.82	1710	0.5

KI: the Kovats retention indices relative to C8-C20 n-alkanes were determined on DB5 capillary column.

**Table 2:** *In vitro* antibacterial activity of *Cinnamomum zeylanicum* bark essential oil.

Microbial strains	Antimicrobial assays			
	Disc diffusion agar (mm)	Well diffusion agar (mm)	MIC (mg/mL)	MBC (mg/mL)
*E. coli*	18.00 ± 0.40	19.00 ± 0.50	6.25	50
*P. aeruginosa*	24.00 ± 0.32	27.00 ± 0.67	3.125	12.5
*S. typhi*	19.00 ± 0.70	22.00 ± 0.48	6.25	25
*L. innocua*	30.00 ± 0.50	34.00 ± 0.46	0.78	3.125
*S. aureus*	26.00 ± 0.44	29.00 ± 0.45	0.78	6.25
*B. cereus*	27.00 ± 0.61	28.00 ± 0.81	1.56	3.125

## Data Availability

All data generated or analyzed during this study are included within the article.
